# Proteomic characterization of the pseudocapsule of clear cell renal cell carcinoma in VHL disease reveals a distinct microenvironment at the tumor boundary zone

**DOI:** 10.1016/j.neo.2025.101214

**Published:** 2025-08-05

**Authors:** Tobias Feilen, Manuel Rogg, Grigor Andreev, Niko Pinter, Maximilian Wess, Anna L. Kössinger, Nastasja Diel, Elke Neumann-Haefelin, Athina Ganner, Markus Grabbert, Christoph Schell, Oliver Schilling

**Affiliations:** a Institute of Surgical Pathology, Medical Center, Faculty of Medicine, University of Freiburg, Freiburg, Germany; b German Cancer Consortium (DKTK) and German Cancer Research Center (DKFZ), Heidelberg, Germany; c Renal Division, Department of Medicine, Freiburg University Medical Center, Faculty of Medicine, University of Freiburg, Freiburg, Germany; d Department of Urology, Medical Center, Faculty of Medicine, University of Freiburg, Freiburg, Germany; e Department II of Internal Medicine, Faculty of Medicine and University Hospital, University of Cologne, Cologne, Germany; f Spemann Graduate School of Biology and Medicine (SGBM), University of Freiburg, Freiburg, Germany; g Faculty of Biology, University of Freiburg, Freiburg, Germany

**Keywords:** Clear cell renal cell carcinoma, Von Hippel-Lindau disease, Pseudocapsule, Extracellular matrix, Proteomics

## Abstract

Von Hippel-Lindau (VHL) disease describes a hereditary tumor predisposition syndrome, caused by germline mutations in the *VHL* tumor suppressor gene, resulting in the functional loss of the VHL protein (pVHL). pVHL loss translates into a pseudo-hypoxic state that drives clear cell renal cell carcinoma (ccRCC) development. ccRCC tumors frequently form a pseudocapsule (PC) at the tumor boundary. This study describes the first comprehensive proteomic analysis of the PC in ccRCC patients with hereditary *VHL* inactivation, revealing a distinctive matrisomal signature. We conducted a deep, mass spectrometry-based proteomic analysis of 130 formalin-fixed paraffin-embedded (FFPE) ccRCC samples, comprising 54 tumor, 45 PC, and 31 non-malignant adjacent tissue (NAT) specimens from 34 patients. The PC exhibited unique matrisomal features, with pronounced enrichment of structural extracellular matrix (ECM) components, ECM processing enzymes, and secreted signaling proteins such as TGFβ2. Its proteome composition, including proteins involved in immune response, varied with tumor size and semi-tryptic peptide analysis indicated selective ECM processing in the PC and elevated levels of proteolysis within the tumor. Further, tumor proteomes reflected canonical *VHL*-driven metabolic reprogramming, including upregulated glycolysis and hypoxia markers, suppressed aerobic metabolism, and dysregulated fatty acid metabolism. Enriched immunoproteasome, MHC-I, and inflammasome proteins indicated an active immune response. Pro-angiogenic factors enriched in the tumor partially extended into the PC. Comparison of primary vs metachronous ccRCC cases uncovered proteomic tumor plasticity in VHL disease. Together, our study delineates the PC as an active, signaling-rich compartment at the ccRCC boundary with potential implications for tumor progression and clinical relevance beyond a mere structural scaffold.

## Introduction

Renal cell carcinoma (RCC) ranks among the ten most common malignancies worldwide. In 2020, over 430,000 individuals were diagnosed with RCC, with males facing approximately twice the risk compared to females [1]. Clear cell RCC (ccRCC), a tumor arising from renal proximal tubular epithelial cells, is the most frequent subtype, accounting for over 70 % of all RCC cases [1]. It occurs either sporadically or in hereditary form in the context of the von Hippel-Lindau (VHL) syndrome, a rare, autosomal-dominant tumor predisposition syndrome responsible for 3 to 5 % of all RCC cases [2]. ccRCC is commonly characterized by the presence of a pseudocapsule (PC), a distinct capsule-like compartment at the interface between tumorous tissue and adjacent non-malignant parenchyma, which is observed in > 90 % of RCC cases [3]. The expansive growth pattern of ccRCC generates compression forces at the tumor boundary, driving the formation of this heterogeneous zone [4]. Histologically, the PC primarily comprises collagen fibers, smooth muscle bundles, and fibrous tissue resulting from compression-induced ischemia and subsequent necrosis. In a clinical context, an intact PC functions as a physical barrier between the tumor and NAT, facilitating nephron-sparing surgeries and is associated with a more localized tumor growth. Conversely, a disrupted, less fibrotic PC correlates with a more aggressive tumor behavior, higher tendency for metastasis, and shorter progression-free survival [[5], [6], [7], [8]]. Despite the potential clinical implications, a comprehensive proteomic analysis specifically targeting the PC compartment remains lacking. At the molecular level, ccRCC is primarily characterized by the loss of the *VHL* tumor suppressor gene, accompanied by secondary inactivating mutations on the remaining allele. *VHL* encodes the pVHL protein, which functions as the substrate recognition component of a multiunit ubiquitin ligase complex (including ELONGIN B/C, CULLIN2, and RBX1) and is involved in the proteasomal degradation of specific target proteins, notably hypoxia-inducible transcription factors (HIF 1α/2α) [9]. *VHL* deficiency results in constitutively activated HIF-mediated gene expression, creating a pseudo-hypoxic state associated with enhanced cell proliferation, survival, and angiogenesis [10]. This state is accompanied by metabolic reprogramming, including elevated anaerobic glucose metabolism, suppression of the tricarboxylic acid (TCA) cycle, decreased oxidative phosphorylation (OXPHOS), and dysregulated fatty acid metabolism [11]. Aside from neo-angiogenesis (via VEGF), HIF-signaling significantly impacts the matrisome, a term collectively describing extracellular matrix (ECM), basement membranes, as well as associated proteins such as proteoglycans and modifying enzymes like proteases [[12], [13], [14]]. There is growing evidence that the tumor-associated matrisome influences numerous cancer hallmarks, including proliferation, evasion of growth suppression, angiogenesis, invasion, metabolic rewiring, and immune evasion [15]. Based on previous studies, we hypothesize that *VHL* loss reshapes the ccRCC matrisome and yields a tumor-promoting microenvironment [16]. VHL syndrome is a rare disease, and only a few omics studies have provided an in-depth proteomic profiling [17]. The majority of published studies have focused on the sporadic disease and primarily investigated tumor and NAT samples, lacking analysis of the PC compartment [[18], [19], [20]]. Therefore, the current study aims to address this gap by providing a comprehensive proteomic characterization of tumor tissue, PC, and NAT in hereditary ccRCC.

## Materials and methods

### Ethics statement

This study was conducted in compliance with the ethical guidelines and received approval from the Ethics Committee of the University Medical Center Freiburg (576/17 and 79/20). All patients provided written informed consent for the use of their tissues for research purposes. Before inclusion, all patient data were pseudonymized.

### Tissue collection, fixation, and dissection

Tissue specimens were collected between 2007 and 2022 during surgical resection of primary and metachronous tumors at the University Medical Center Freiburg. After resection, tissues were transferred into formalin solution. Tissue specimens were sectioned, processed, and analyzed following standard protocols for histopathological diagnostics, including embedding in paraffin. For proteomic analysis, 10 µm thick tissue slices were automatically deparaffinized and dissected guided by corresponding H&E stainings in NAT, PC, and tumor compartments by an experienced pathologist. Representative H&E images of the tumor, PC and NAT are provided in Fig. S1.

### Sample preparation for LC-MS/MS analysis and data acquisition

Tissue specimens were transferred into 1.5 mL tubes containing 150 µL lysis buffer (100 mM HEPES, 1 % (w/v) SDS, pH 8), followed by heat-induced antigen retrieval (HIAR; 2 h, 95°C) and ultra-sonication in a Bioruptor Plus for 20 cycles á 30 s (Diagenode, Belgium). Protein concentrations were estimated using the bicinchoninic acid (BCA) assay (Thermo Fisher Scientific, Germany), yielding 50–150 µg of protein. 40 µg of protein was transferred into a 96-deepwell plate, and the volume was adjusted to 150 µL with lysis buffer. All subsequent sample processing steps were performed using the automated liquid handling platform AssayMAP Bravo (Agilent Technologies, USA). Proteins were reduced and alkylated with 5 mM TCEP and 20 mM CAA for 30 min at 37°C in the dark. Proteins were enriched on magnetic beads using the previously published SP3 protocol [21]. Briefly, Sera-Mag magnetic carboxylate beads (Cytiva, USA) were added (3.1 µg/µL), followed by acetonitrile (ACN) addition (60 % v/v final concentration) and shaking (10 min, 1050 rpm). Detergents and buffer were removed by 3 washes (2x EtOH 70 %, 1x ACN 100 %). Beads were resuspended in ABC buffer (100 mM, pH 8), followed by a tandem digestion using lysyl endopeptidase (Lys-C, Wako Chemicals, Germany) and trypsin (Serva, Germany). For Lys-C digestion, a 1:50 (w/w) protease:protein ratio was employed at 42°C, 800 rpm for 2 h. Subsequently, trypsin digestion was carried out using a 1:25 (w/w) protease:protein ratio at 37°C, 800 rpm for 15 h. After digestion, beads were separated from the supernatant using a magnetic rack and centrifugation (21.800 rcf, 10 min). Samples were vacuum dried at 45°C. After reconstitution in 60 µL H_2_O, peptide concentrations were estimated using the peptide BCA assay (Thermo Fisher Scientific, Germany).

### LC-MS/MS measurement

Peptides were diluted to 25 ng/µL in 0.1 % formic acid and spiked with 100 fmol of eleven synthetic indexed retention time (iRT) peptides. 500 ng per sample were loaded onto Evotips (Evosep, Denmark) following the manufacturer’s protocol. LC-MS/MS measurements were performed on a TimsTOF Flex Mass Spectrometer (Bruker Daltonics, Bremen, Germany) equipped with a CaptiveSpray ion source. Chromatographic separation was conducted using the Evosep One HPLC system (30SPD method: 44 min gradient, 500 nL/min) and the EV1137 performance 15 cm reversed-phase column (Evosep). Buffer A: 0.1 % aqueous formic acid, buffer B: 0.1 % formic acid in ACN. The mass-spectrometer was operated in positive dia-PASEF mode with a 100–1700 *m*/*z* range and 0.7–1.3 V*s/cm^2^ ion mobility (1/K0) range. Collision-induced dissociation energy was set to 20–59 eV, ion accumulation time to 100 ms with a 100 % duty cycle. The capillary voltage was set to 1600 V and the drying gas flow rate was 3 L/min (180°C drying temperature). For DIA measurements, 50 mass windows with optimized *m*/*z* range were applied, giving a total cycle time of 2.76 s.

### Data analysis

Raw LC-MS/MS data were processed using DIA-NN (v1.9.2) [22,23]. A predicted spectral library was constructed from the human reference proteome database (downloaded from https://www.ebi.ac.uk/reference_proteomes/ on 22/07/2023), plus common contaminants and 11 iRT peptides. The match between runs (MBR) algorithm was applied, refining the predicted library across all cohort samples. Peptide precursors within a range of 7–30 amino acids and charge states of 1–4 were included, with an *m*/*z* window of 100–1700 and allowance of one missed cleavage. Fragment ion *m*/*z* ranged from 390 to 1210, and data were filtered at a false discovery (FDR) threshold of 1 %.

Statistical and exploratory analyses were conducted in RStudio (v4.3.0) using in-house scripts and publicly available R packages. Quantitative proteomic data were extracted using the DIA-NN R package (v1.0.1), applying the MaxLFQ algorithm to calculate protein abundances exclusively from proteotypic peptides [22,24]. Protein intensities were log2 transformed prior to analysis. The mixOmics R package (v6.24.0) was utilized for supervised and unsupervised explorative statistics [25]. Differential abundance analysis was performed using Limma (v3.56.2), and gene set enrichment analysis was carried out via the clusterProfiler package (v4.8.3), incorporating Gene Ontology databases [[26], [27], [28]]. Missing data were imputed using the DIMA package (v0.4.18) [29]. For batch effect correction and alignment of datasets, HarmonizR (v1.0.0) was applied, and ECM proteins were classified via MatrisomeAnalyzeR (v1.0.1) [30]. For co-expression analysis, we applied the Python-based Clust algorithm [31].

### Immunohistochemistry

Immunohistochemistry (IHC) staining of representative ccRCC samples encompassing tumor, PC, and NAT was performed as recently described [32]. In brief, 2 µm sections of FFPE tissue were generated. HIAR was then performed in Tris-EDTA buffer (pH 9) for MMP14 (MAB3328, Merck Millipore), and PDGFRB (3169, Cell Signaling) or citrate buffer (pH 6) for COL1A1 (72026, Cell Signaling), CD45 (IR751, Dako), and panCK (IR03, Dako) staining. Primary antibodies were incubated overnight. IHC staining was subsequently performed using the DAB chromogen system.

## Results and discussion

### Patient cohort overview and proteome coverage

**Fig. 1:**
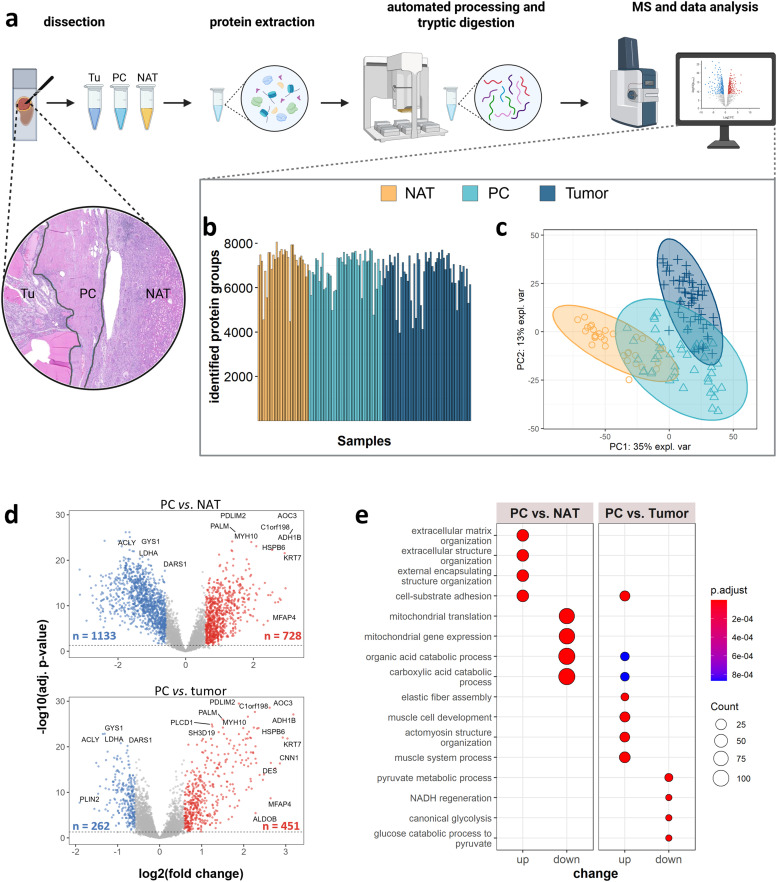
**Syndromic ccRCC cohort**: **a** Overview of experimental workflow, including tissue dissection, separating NAT, PC, and tumor, protein extraction, automated sample processing with tryptic digestion, followed by MS measurement and data analysis. **b** Identified protein groups across all samples of the cohort. **c** Principal component analysis (PCA) of tumor, PC, and NAT samples. Ovals indicate 95 % confidence intervals. **d** Volcano plots showing differentially regulated proteins between PC and NAT (top) and PC and tumor (bottom). Proteins on the right are upregulated in the PC (fold change ≥ |1.5|, BH adj. p-value ≤ 0.05). **e** GO enrichment analysis of upregulated and downregulated biological processes in PC vs NAT and PC vs tumor (BH adj. p-value ≤ 0.05). Created with Biorender.com.

In this study, we aimed for a comprehensive proteomic analysis of ccRCC in patients with VHL syndrome, based on the corresponding registry of the University Medical Center Freiburg. In total, 130 samples, comprising 54 tumor, 45 PC, and 31 NAT specimens from 34 patients were analyzed, including samples from 12 patients who developed up to three metachronous tumors (i.e., tumor developing after initial resection of the first diagnosed ccRCC). The cohort comprised 14 male and 20 female patients with average ages of 36 years (interquartile range IQR = 32–42) and 46 years (IQR = 46–59), respectively. The majority of tumors (*n* = 40) were classified as grade 2 (G2), while 11 tumors were G1 and 3 tumors G3, respectively. The distribution of the affected kidney (left or right) was evenly balanced. Syndromic *VHL* mutations comprise deletions, missense, nonsense, frameshift, and splice variants. Further cohort information can be found in Table S1. The study was conducted using FFPE tissue specimens, as shown previously (Fig. 1a) [33]. Across all samples, we identified a total of 9,147 protein groups at a 1 % false discovery rate (FDR), with an average of 7022 ± 892 protein groups per sample (Fig. 1b). This high level of proteome coverage was supported by the application of the py_diAID algorithm, which increased protein group identifications by up to 20 % compared to non-optimized measurements from a master mix of the samples [34]. Our deep proteome coverage allowed for the stringent missing data filtering, including only proteins with ≥ 70 % completeness in each condition, yielding a robust dataset containing 5,614 proteins. However, as our study comprises inherently different entities with potential tissue-specific expression patterns (tumor, PC, and NAT), we applied a second filtering criterion. This included proteins detected in ≥ 50 % of samples within any of the three conditions, expanding the dataset to 7,719 proteins. Missing values in the extended dataset were imputed to enable reliable differential abundance analyses.

To assess whether our proteome analysis of ccRCC is representative of the disease, we integrated a publicly available CPTAC proteome dataset using a ComBat-based batch correction [18,35]. PCA demonstrated a clear overlap of tumor and NAT samples from both datasets (Fig. S2), highlighting that our data on syndromic ccRCC represents prototypical ccRCC proteome biology and further corroborates the findings from our study.

### Proteomic signature of the ccRCC pseudocapsule (PC)

Despite the potential impact of the PC on tumor progression and metastasis, no proteomic study has yet focused on its characterization in ccRCC to date. To address this gap, we conducted the proteomic profiling of 45 dissected PC specimens. Principal component analysis (PCA; Fig. 1c) and Uniform Manifold Approximation and Projection (UMAP; Fig. S3) clearly separated the tumor and the corresponding NAT samples, while PC proteomes clustered in between and partially overlapped with both tumor and NAT.

**Fig. 2:**
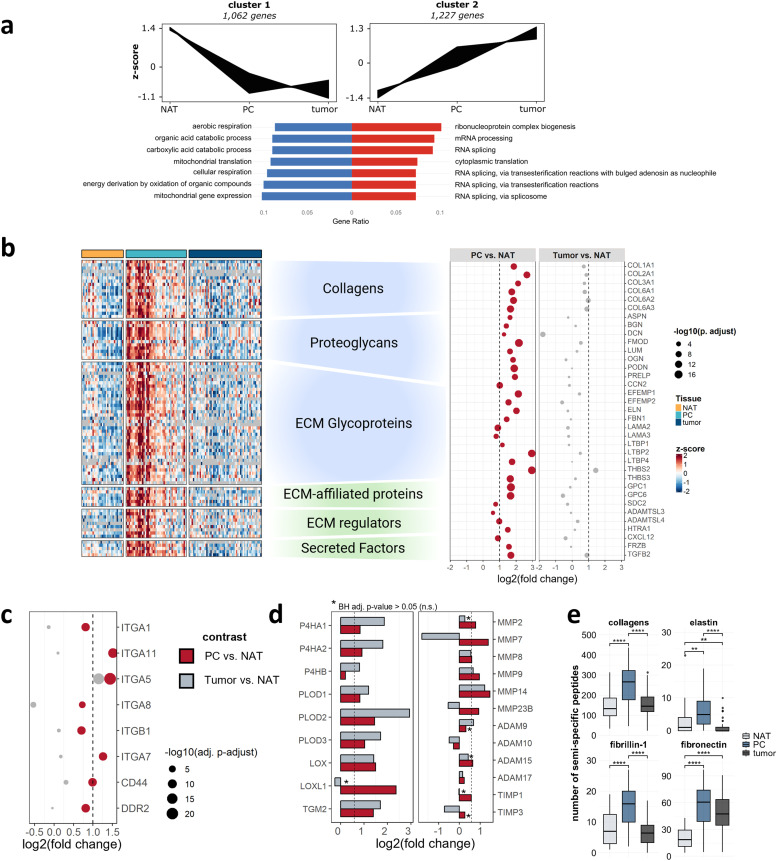
**The matrisomal landscape of the PC: a** Top: Co-expression analysis reveals 2 distinct gene clusters with opposite trends in abundance from NAT to PC to tumor. Bottom: GO enrichment analysis of upregulated biological processes within gene cluster 1 (blue) and cluster 2 (red). **b** Heatmap (left) comparing the abundance of PC enriched matrisomal proteins and dotplot (right) showing the log2 fold changes of selected matrisomal proteins in PC and tumor vs NAT. **c** Dotplot of selected significantly upregulated integrins and other ECM receptors in PC vs NAT (imputed dataset, fold change ≥ 1.5, BH adj. p-value ≤ 0.05) **d** Barplot showing log2 fold changes of selected ECM synthesis and remodeling proteins (imputed dataset, BH adj. p-value ≤ 0.05, *n.s.) **e** Boxplots showing the number of identified semi-specific peptides of selected ECM scaffolding proteins; **p < 0.01; ****p < 0.0001 (Dunn’s test, Tukey’s test). Created with Biorender.com.

This intermediate clustering indicates that the PC shares features with both tissues, while also exhibiting a unique biology. Differential abundance analysis confirmed significant proteomic differences between the PC and both tumor and NAT (Fig. 1d). These compartment-specific characteristics are likewise evident at the histological level, as illustrated by IHC in Fig. S4. Compared to NAT, the PC proteome was notably enriched in matrisomal proteins involved in ECM organization and cell-substrate adhesion (Fig. 1e). In contrast, proteins related to mitochondrial translation exhibited a marked downregulation in the PC relative to NAT. When compared to tumor tissue, the PC revealed decreased levels of proteins involved in pyruvate metabolism and glycolysis. Complementing cancer-focused gene set enrichment analysis based on the Curated Cancer Cell Atlas (3CA) revealed a pronounced fibrotic fingerprint in the PC, alongside metabolic suppression and hypoxia (Fig. S5). In line with the PCA results, co-expression analysis identified two major protein clusters with gradual changing abundance across tissues (Fig. 2a) [31]. Proteins in cluster 1 exhibited a gradual decline in abundance from NAT to PC to tumor and were primarily linked to mitochondrial gene expression, oxidative metabolism, and cellular respiration. By contrast, cluster 2 proteins showed a gradual increase across tissues and were mainly associated with ribonucleoprotein complex biogenesis and mRNA synthesis. This gradual shift in abundance may reflect the differing cellular composition of the three compartments. Collectively, these findings suggest that the PC integrates proteomic features of both non-malignant and cancerous tissues, while exhibiting reduced metabolic activity.

### Proteins involved in ECM remodeling and fibrotic signaling characterize the PC proteome

Beyond co-expression analysis, differential abundance analysis identified 265 uniquely enriched and 48 depleted proteins in the PC relative to both tumor and NAT (Fig. S6). Strikingly, one-third of the upregulated proteins were classified as matrisomal, indicating a pronounced ECM signature. An overview of the PC-specific matrisome is presented in Fig. 2b. The PC matrisome was particularly enriched in a broad spectrum of collagens, including COL1A1 and COL6A1, consistent with recent findings [36]. Additional ECM scaffolding glycoproteins (ELN, FBN1, and laminins; Fig. 2b), as well as integrins and other ECM receptors (CD44, DDR2; Fig. 2c) showed likewise high abundance in the PC, pointing towards distinct cell-ECM adhesion and signal transduction [37,38]. Moreover, we observed elevated levels of small leucine-rich proteoglycans (e.g., ASP, BGN, DCN) and syndecan-2 (SDC2), playing crucial roles in collagen fibril formation, stabilization, and modulation, reflecting the presence of a pro-fibrotic microenvironment [39]. Proteolytic ECM regulators, including members of the ADAMTSL protein family and HTRA1, indicated active ECM turnover through modulation of fibrillin assembly and elastic fiber formation. We further identified extracellular regulators of growth factor bioavailability, including glypicans (GPC1, GPC6) and latent-TGFβ-binding proteins (LTBPs), suggesting controlled bioavailability and activation of growth factors, such as TGFβ, within the PC [[40], [41], [42], [43], [44]]. By contrast, many collagen assembly and crosslinking enzymes, including prolyl and lysyl hydroxylases, were differentially regulated (Fig. 2d) and, except for LOXL1, more abundant in the tumor vs PC, suggesting increased collagen processing in the tumor [45]. Interestingly, we noticed elevated levels of matrix metalloproteinases (MMPs), surpassing a much milder increase of their endogenous inhibitors (TIMPs) and pointing to dynamic ECM processing and the potential release of growth factors and bioactive protein fragments [46]. Notably, the pro-fibrotic MMP7 shows pronounced inverse regulation, with reduced abundance in the tumor, while upregulated in the PC. Consistent with this, increased numbers of semi‐specific (i.e., proteolytically truncated) collagen (e.g., COL1A, COL6A, COL14A chains), elastin, fibrillin-1, and fibronectin peptides further indicated ongoing ECM processing in the PC (Fig. 2e). Notably, 75 % of the identified semi-specific collagen peptides originated from COL1A and COL6A chains.

**Fig. 3:**
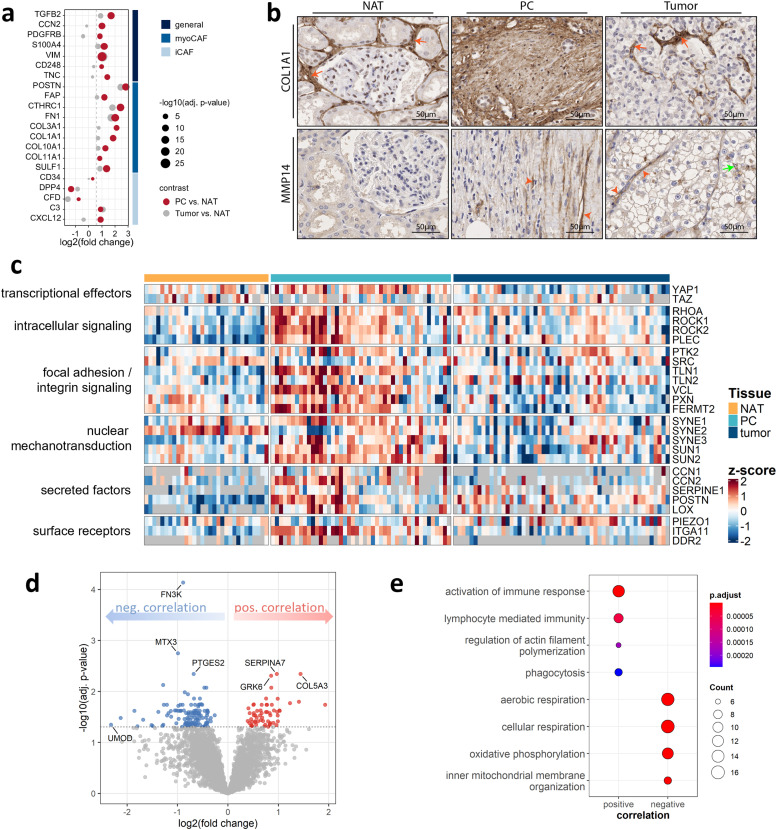
**Mechanotransduction and tumor size correlation analysis: a** Dotplot of selected significantly upregulated general fibrosis-associated proteins, markers for myofibroblast-like cancer-associated fibroblasts (myoCAF) and inflammatory CAFs (iCAF) in the PC vs NAT (imputed dataset, fold change ≥ 1.5, BH adj. p-value ≤ 0.05). **b** Immunohistochemistry analysis of COL1A1 and MMP14 of representative ccRCC samples encompassing tumor, PC, and NAT. Orange arrows denote pericapillary deposition of COL1A1 by interstitial cells. Orange arrowheads indicate endothelial expression of MMP14, while green arrows indicate MMP14-positive mesenchymal cells. **c** Heatmap comparing the abundance of selected mechanotransduction proteins in tumor, PC, and NAT. **d** Volcano plot showing PC proteins with positive (red) a negative (blue) correlation with tumor size (BH adj. p-value ≤ 0.05). **e** GO enrichment analysis of biological processes in PC proteins with positive and negative correlation with tumor size.

In addition, the PC proteome was markedly enriched in key fibrotic drivers such as TGFβ2 and CCN2, alongside several fibroblast-associated proteins including fibroblast-specific protein 1 (S100A4), platelet derived growth factor receptor beta (PDGFRB) and CD248, together with a panel of cancer-associated fibroblast (CAF) subtype markers (Fig. 3a) [47,48]. Stratification of these markers revealed a pronounced accumulation of myofibroblast-like CAF (myoCAF) proteins, such as POSTN, FAP FN1 and different collagens (e.g., COL1A1/3A1), whereas the inflammatory CAF (iCAF) markers were only mildly enriched (C3, CXCL12) or even depleted (DPP4, CFD). These observations reinforce the predominance of contractile myoCAFs in the PC and underscore their role in shaping this unique microenvironment. Taken together, our data suggest that the PC represents a fibrotic and signaling-rich interface shaped by fibroblasts, augmented ECM deposition and remodeling, as well as enhanced growth factor signaling and thus indicates that the PC’s role extends beyond that of a mere structural scaffold.

Immunohistochemical analysis further substantiated our proteomic findings, emphasizing distinct expression patterns of COL1A1 and MMP14 in the PC relative to NAT and tumor tissue (Fig. 3b). COL1A1 staining revealed pericapillary deposition by interstitial cells in NAT and tumor. In accordance with our mass spectrometry data, which suggests elevated ECM deposition and remodeling, a more pronounced and widespread staining of COL1A1 was observed across the entire PC compartment. For MMP14, immunostainings demonstrated an endothelial localization within both PC and the tumor. Conversely, NAT showed no detectable MMP14 expression.

### Mechanotransduction and tumor size-dependent alterations of the PC proteome

The PC in ccRCC is thought to arise upon tumor expansion and subsequent compression and necrosis of the adjacent renal parenchyma. Therefore, we profiled mechanotransduction-associated proteins in NAT, PC, and tumor (Fig. 3c) and found distinct mechanotransduction signatures in the PC. While the transcriptional effectors YAP1 and TAZ exhibited only minimal and no PC-specific enrichment, respectively, we observed prominent enrichment of intracellular signaling proteins, notably RHOA and ROCK family kinases. Likewise, proteins associated with focal adhesion and integrin signaling, such as PTK2 (focal adhesion kinase 1, FAK), talin-1, and vinculin, demonstrated elevated levels in the PC, suggesting active mechanical linkage and signal transduction. Additionally, proteins implicated in nuclear mechanotransduction, specifically nesprins (SYNE1, SYNE3) and SUN domain proteins (SUN1, SUN2), exhibited enrichment in the PC, concomitant with proteins that are commonly secreted in response to mechanical stress (CCN2, POSTN) and the surface mechanosensors ITGA11 and DDR2. In consideration of the observed mechanotransduction signatures, we examined whether progressive tumor growth and the potentially associated mechanical stress on the PC reshapes its proteomic landscape. Previous studies have linked tumor size to changes in the PC composition [3]. Using linear correlation analysis, we related tumor diameter to protein abundance within the PC and detected 62 proteins whose levels rose and 117 whose levels fell as tumors enlarged (Fig. 3d). These tumor size-dependent shifts emphasize the impact of tumor-induced mechanical stress on the PC proteome. Proteins exhibiting a positive correlation were primarily involved in activation of immune response (e.g., complement components, caspase-1, CD14, and CD163) (Fig. 3e, S7). Conversely, proteins showing a negative correlation were associated with aerobic metabolic processes (Fig. 3e, S8), potentially reflecting a shift towards a more anaerobic or hypoxic microenvironment in PCs of larger tumors.

### Proteome profiling reveals metabolic reprogramming, proliferation, angiogenesis, and immune activation in ccRCC

**Fig. 4:**
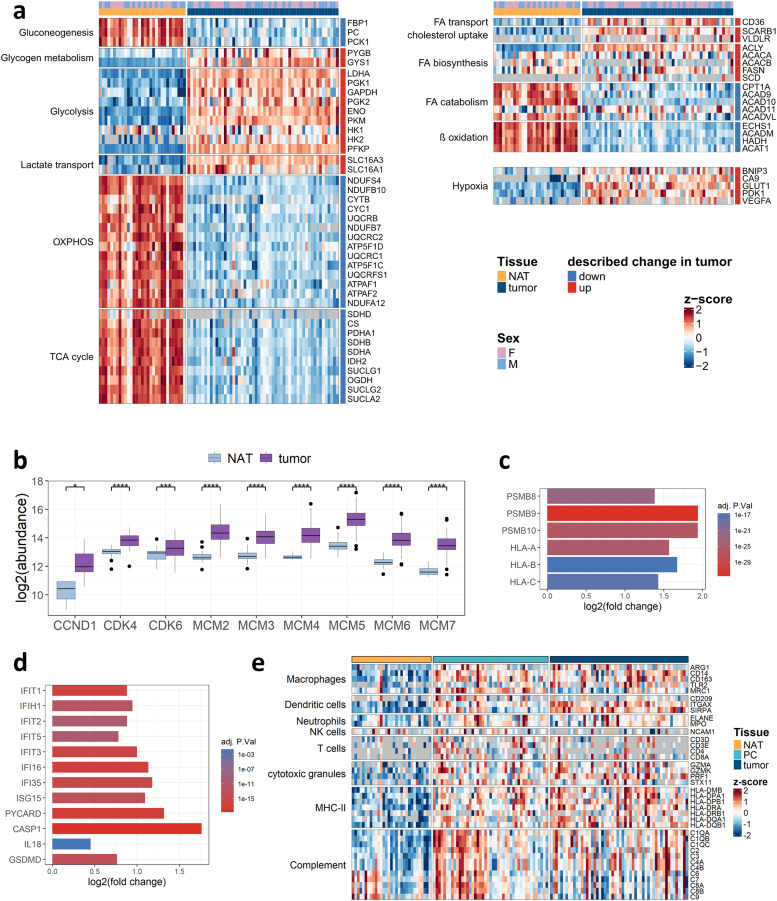
**The ccRCC tumor landscape: a** Heatmap comparing the abundance of selected metabolic proteins, commonly dysregulated in ccRCC tumors vs NAT. **b** Boxplot comparing the abundance of selected cell-proliferation marker proteins; *p < 0.05; ***p < 0.001; ****p < 0.0001 (t-test, Mann–Whitney U test). **c&d** Barplot of significantly upregulated proteins associated with immunoproteasome (**c**) and inflammasome (**d**). **e** Heatmap comparing the abundance of proteins associated with the presence of immune cells, antigen presentation and complement activity in tumor, PC, and NAT.

**Fig. 5:**
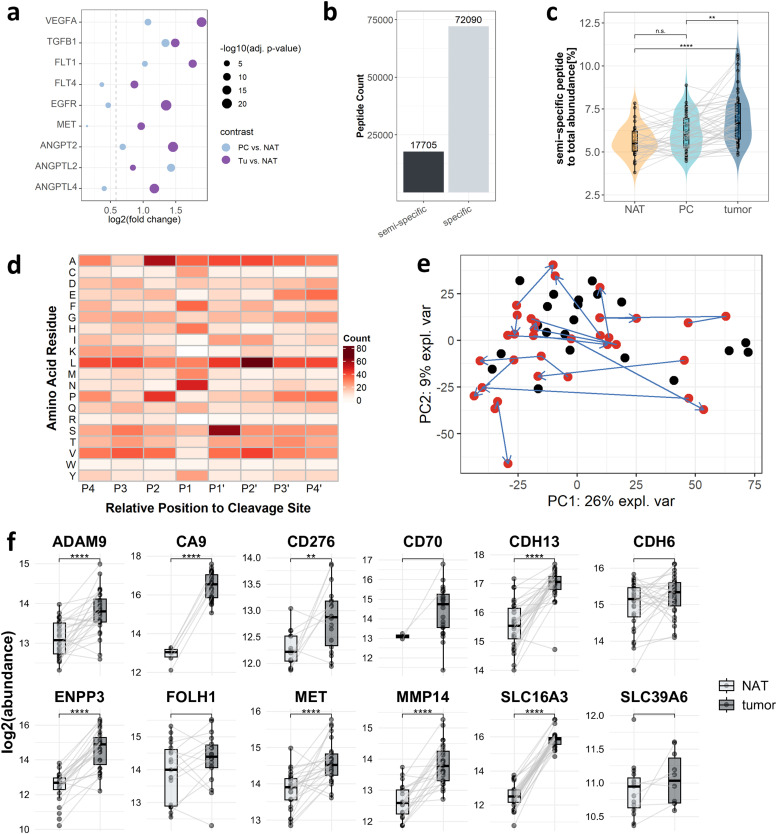
**Semi-specific peptide analysis and ADC target candidates: a** Dotplot of selected significantly upregulated angiogenic proteins (imputed dataset, fold change ≥ 1.5, BH adjusted p-value ≤ 0.05) **b** Barplot showing the number of identified semi-specific and specific peptides **c** Boxplot comparing the relative abundance of semi-specific peptides between tumor, PC and NAT, **p < 0.01; ****p < 0.0001 (Dunn test, Tukey test). **d** Heatmap showing proteolytic patterns of upregulated proteolytic products. **e** PCA showing only tumor samples. Cases of patients with one or more metachronous tumors are highlighted in red and connected by arrows. **f** Boxplot showing the log2 abundance of selected ADC target candidates in tumor and NAT. Grey lines indicate patient-matched tumor and NAT samples; **p < 0.01; ***p < 0.001; ****p < 0.0001 (t-test, Mann–Whitney U test).

Consistent with previous studies, our tumor proteome analysis revealed that ccRCC tumors are characterized by a pronounced upregulation of glycolytic and hypoxia-associated proteins compared to NAT (Fig. 4a) [10,11]. This is accompanied by a marked depletion of aerobic metabolic pathways, such as TCA cycle and OXPHOS; together with a dysregulation of the fatty acid metabolism, likely contributing to intracellular lipid accumulation. Beyond metabolic reprogramming, we noted a significant enrichment of cell cycle and DNA replication proteins, including cyclin-dependent kinases (CDK4 and CDK6), cyclin D1 (CCND1), and the minichromosome maintenance complex proteins (MCM2–7; Fig. 4b), in line with increased cellular proliferation rates [49]. Since the canonical proliferation marker Ki-67 was not detected in our dataset, we correlated the abundance of the proliferating cell nuclear antigen (PCNA) with our set of DNA replication proteins in tumor and PC (Fig. S9). PCNA showed a strong positive correlation with MCM2–7 in tumor and PC (*r* = 0.49–0.67), but only weak associations with CCND1, CDK4, and CDK6, thereby validating the MCM2–7-based proliferation signature. Findings pointing towards an elevated immune response include the enrichment of the immunoproteasome (PSMB8–10) and major histocompatibility complex class I (MHC-I) proteins (Fig. 4c). In addition, we observed significant tumor enrichment of the IFI16 inflammasome pathway comprising the sensor protein IFI16, the adaptor PYCARD, caspase-1 and its substrate gasdermin-D (Fig. 4d). Concurrently, interferon-induced proteins (e.g., ISG15, IFIT1-3, and IFI35) were also significantly enriched in the tumor, collectively pointing to active interferon signaling and inflammasome-mediated processes. Additionally, enriched markers for macrophages (e.g., ARG1, CD14, and CD163), dendritic cells (CD209, ITGAX, and SIRPA), and neutrophils (ELANE, MPO) in both tumor and PC suggest an activated innate immune response. Concomitantly, cytotoxic granule proteins (e.g., GZMA, GZMK, and PRF1) displayed higher abundance in both tumor and PC. Although T-cell markers (e.g., CD4 and CD8A) were relatively sparse, they were likewise elevated in these regions, suggesting T-cell activity and increased adaptive immune response. Moreover, the upregulation of MHC-II and complement proteins points towards active antigen presentation and complement-mediated immune response. To directly link proliferation with immune infiltration, we generated compartment-resolved correlation matrices between PCNA and a curated panel of myeloid- and lymphoid-cell markers (Fig. S10). In tumor regions, PCNA abundance correlated most strongly with ITGAX, ELANE, MPO, CD3E, and CD8A, indicating that infiltrating myeloid and T cells contribute to the composite proliferation signature alongside tumor cells. Conversely, in the PC, only SIRPA showed a comparable correlation with PCNA, while correlations with the remaining markers were weak or negative. Employing differential abundance analysis on the imputed dataset, we further observed the significant enrichment of pro-angiogenic growth factors and receptors (e.g., VEGFA, FLT1/VEGFR-1, EGFR, and MET), members of the pro-angiogenic angiopoietin family (ANGPT2, ANGPTL2, and ANGPTL4), and other growth factors (TGFβ1). These angiogenic signatures were especially pronounced in the tumor and, with a more moderate presence, in the PC (Fig. 5a). Collectively, these observations indicate that ccRCC tumors of VHL patients exhibit a proteome fingerprint defined by hypoxia-driven metabolic reprogramming, proliferative signaling, angiogenesis, and immune‐mediated activity. Recent studies have emphasized that peptide-level comparisons may uncover domain-specific alterations of proteins in a given condition; hence, deepening insight into proteome biology beyond proteome abundance [50]. As a first step towards applying this concept, we investigated the correlation between differential peptide vs protein abundance in the tumor vs NAT comparison. We observed a strong overall correlation between protein-level and tryptic peptide-level log_2_ fold changes, substantiating the robustness of our quantitative analysis (Fig. S11). However, we observe peptides that exhibit significant changes where the corresponding proteins showed either no or opposite regulation (e.g., KRT14, COL6A3, and PFKM).

### Distinct proteolytic processing in NAT, PC, and tumor

To gain a deeper insight beyond the database search restricted to fully tryptic peptides, we performed a semi-specific peptide analysis to identify peptides that had undergone endogenous proteolytic processing prior to tryptic digestion [51]. This analysis identified a total of 17,705 semi-specific peptides, accounting for 19.7 % of the total identified peptides and representing between 8.9 % and 16.0 % of peptides per sample (Fig. 5b). Notably, peptides including the annotated protein N- or C-terminus were excluded from the analysis to ensure the focus on endogenous proteolytic processes. Investigating the relative abundance of semi-specific peptides, we observed that the proportional intensity of semi-specific peptides was significantly elevated in the tumor compared to both PC and NAT (Fig. 5c), suggesting elevated proteolytic activity within the tumor. Differential abundance analysis revealed significantly upregulated peptides within the tumor vs NAT. However, subsequent cleavage motif analysis of significantly upregulated peptides did not allow for an unambiguous conclusion regarding the likely involved proteases (Fig. 5d).

### Metachronous ccRCC tumors display proteomic plasticity

When examining tumor samples alone, PCA revealed that patient-matched primary and metachronous tumors did not cluster together, hence suggesting proteomic variability and plasticity (Fig. 5e). We consider it likely that genomic divergence between metachronous tumors of the same patient strongly contributes to the observed proteomic plasticity. However, tumor-specific genomic data has remained beyond the reach of the present study. In view of this limitation, we resorted to the surrogate of proteomic markers that have been linked to genomic aberrations in previous studies [18,19,52]. Clark et al. showed that low PBRM1 abundance marks either PBRM1 mutation or gene methylation in ccRCC [18]. Comparing the matched metachronous tumor proteome profiles, we observe a wide spectrum of PBRM1 levels, including (i) “metachronous loss” (high in the primary, low/absent in one or more metachronous tumors); (ii) “metachronous gain” (low in the primary, higher in metachronous lesions); and (iii) similar levels in both primary and metachronous tumors (Fig. S12a). Li et al. suggested that BAP1 mutations yield increased UCHL1 levels [19]. On a cohort-wide level, our data supports an inverse relation of BAP1 and UCHL1 (Fig. S12b). Yet, considerable variability of the expression data renders comparison of individual, patient-matched samples inconclusive. Li et al. associated IGF2BP3 and PYCR1 overexpression with genome instability [52]. For our data, we considered expression of the mismatch-repair proteins MLH1, MSH2, and MSH6 as a surrogate for genome (in-)stability. Their expression does not correlate with the levels of IGF2BP3 or PYCR1. This divergence became further apparent for investigational antibody-drug conjugate (ADC) target proteins (Fig. 5f). The patient-matched protein abundance revealed an overall trend towards higher abundances in tumor tissue, but individual patient samples showed considerable variation. For some cases, the abundance of investigational target proteins (CDH6, MET, and ADAM9) decreased in the tumor relative to NAT, emphasizing the heterogeneity of ADC target abundance across patients and metachronous cases.

These findings indicate that, while many ccRCC tumors exhibit increased levels of therapeutically relevant proteins, not all follow this pattern, underscoring the need for individualized proteomic profiling of tumors when considering future ADC‐based treatment strategies in sporadic and hereditary ccRCC.

## Conclusion

In summary, our comprehensive proteomic analysis of 130 FFPE tissue samples, including tumor, PC, and NAT, from 34 ccRCC patients with VHL syndrome has yielded a deep proteome coverage, identifying over 9,100 protein groups, allowing for significant insights into the disease's molecular landscape. Co-expression analysis revealed two distinct protein clusters with a gradual changing abundance. Proteins decreasing from NAT to tumor were primarily involved in aerobic respiration, whereas proteins with increasing abundance were linked to mRNA processing. Notably, the PC uniquely displayed the enrichment of matrisomal proteins involved in ECM deposition, remodeling, and fibrosis, alongside signatures of metabolic suppression, consistent with lower proliferation and metabolic activity. PC-enriched proteins involved in mechanotransduction indicated exposure to elevated mechanical stress. Correlation analysis with tumor size highlighted progressive hypoxia within the PC as the tumors expand. In contrast, tumor proteomes demonstrated canonical hallmarks associated with *VHL* loss, including elevated glycolysis and hypoxia-associated proteins, suppressed aerobic metabolic pathways, and altered fatty acid metabolism. These metabolic shifts were accompanied by significant enrichment of immunoproteasome components, inflammasome-related proteins, and innate and adaptive immune cell markers. Enriched proliferation markers and pro-angiogenic factors reflected the proliferative phenotype of the tumor. Semi-specific peptide analysis provided deeper insight into tissue-specific proteolytic processes and indicated heightened proteolysis in the tumor, whereas PC-specific enrichment of ECM scaffolding peptides pointed to active ECM turnover. Moreover, the notable proteomic plasticity among primary and metachronous tumor lesions within individual patients underscored tumor heterogeneity, which was further evident from the variability in investigational ADC target protein abundance. This heterogeneity emphasizes the necessity for personalized molecular profiling when considering targeted therapeutic strategies. Collectively, our findings offer comprehensive molecular insights into VHL disease-associated ccRCC, highlighting the PC’s critical role beyond that of a mere fibrotic scaffold surrounding the tumor. It exhibits a unique ECM signature, extensive remodeling, pronounced fibrosis, and an enriched set of signaling proteins mediating growth factor signaling and angiogenesis. Further molecular profiling of the PC will be essential to fully elucidate the mechanisms underlying tumor invasion into the PC and its broader implications in tumor progression and metastasis.

### Data availability

The raw spectral data cannot be made publicly available for medical data protection reasons, as it is considered sensitive data. Likewise, peptide and protein expression values are considered sensitive and only reported in an aggregated form [53]. The patient data collected for this study cannot made publicly available due to ethical, privacy and legal considerations. This approach is in line with the underlying patient consent.

## Funding statements

OS acknowledges funding by the 10.13039/501100001659Deutsche Forschungsgemeinschaft (DFG, projects 446058856, 466359513, 444936968, 405351425, 431336276, 431984000 (SFB 1453 “NephGen”), 441891347 (SFB 1479 “OncoEscape”), 423813989 (GRK 2606 “ProtPath”), 322977937 (GRK 2344 “MeInBio”)) 507957722, the ERA PerMed program (BMBF, 01KU1916, 01KU1915A), the German Consortium for Translational Cancer Research (project Impro-Rec), the MatrixCode research group, FRIAS, Freiburg, the investBW program BW1_1198/03, the ERA TransCan program (projects 01KT2201, “PREDICO”, 01KT2333 „ICC-STRAT“), the BMBF KMUi program (project 13GW0603E, project 10.13039/100005634ESTHER), and the BMBF Cluster4Future program (nanodiag). CS acknowledges funding by the German Research Foundation (DFG – Deutsche Forschungsgemeinschaft): SFB1453 (project-ID 431984000); SFB1160 (project-ID 256073931) and the Heisenberg program (project-ID 501370692), further support by the Wilhelm Sander-Stiftung (project-ID 2023.010.1). ENH and AG were supported by the Deutsche Forschungsgemeinschaft: SFB1453 (project-ID 431984000).

## Declaration of generative AI in scientific writing

During the preparation of this work the author(s) used ChatGPT (OpenAI) in order to refine the scientific writing process, finding synonyms and different phrasings. After using this tool/service, the author(s) reviewed and edited the content as needed and take(s) full responsibility for the content of the publication.

## CRediT authorship contribution statement

**Tobias Feilen:** Writing – original draft, Visualization, Methodology, Investigation, Formal analysis, Conceptualization. **Manuel Rogg:** Writing – review & editing, Investigation. **Grigor Andreev:** Writing – review & editing, Investigation. **Niko Pinter:** Writing – review & editing. **Maximilian Wess:** Writing – review & editing. **Anna L. Kössinger:** Writing – review & editing, Data curation. **Nastasja Diel:** Investigation. **Elke Neumann-Haefelin:** Writing – review & editing, Resources. **Athina Ganner:** Writing – review & editing. **Markus Grabbert:** Writing – review & editing. **Christoph Schell:** Writing – review & editing, Resources, Conceptualization. **Oliver Schilling:** Writing – review & editing, Writing – original draft, Supervision, Resources, Investigation, Funding acquisition, Conceptualization.

## Declaration of competing interest

The authors declare that they have no known competing financial interests or personal relationships that could have appeared to influence the work reported in this paper.

## Appendix Supplementary materials

Image, application 1

Image, application 2
