# Supplementary material for: Proteomic characterization of the pseudocapsule of clear cell renal cell carcinoma in VHL disease reveals a distinct microenvironment at the tumor boundary zone
Source: Neoplasia. 2025 Aug 5;68:101214. doi: 10.1016/j.neo.2025.101214 (PMC12446972; doi:10.1016/j.neo.2025.101214)
Supplement: Supplementary file 1 [file mmc1.docx]

# Supplementary Table Legends

**Table S1.** Clinical annotation of syndromic ccRCC cases cohort

| **Variable** | **N** | **Overall**, N = 54*^1^* | **F**, N = 31*^1^* | **M**, N = 23*^1^* | **p-value***^2^* |
| --- | --- | --- | --- | --- | --- |
| **Clinical Data** |  |  |  |  |  |
| Age at Diagnosis  (median, IQR) | 54 | 46 (33, 54) | 53 (46, 59) | 36 (32, 42) | <0.001 |
| **Chronology** | 54 |  |  |  | >0.9 |
| primary |  | 28 (52%) | 16 (52%) | 12 (52%) |  |
| metachronous |  | 26 (48%) | 15 (48%) | 11 (48%) |  |
| **Tumor Grade** | 54 |  |  |  | 0.5 |
| G1 |  | 11 (20%) | 8 (26%) | 3 (13%) |  |
| G2 |  | 40 (74%) | 22 (71%) | 18 (78%) |  |
| G3 |  | 3 (5.6%) | 1 (3.2%) | 2 (8.7%) |  |
| ***VHL* Mutation** | 54 |  |  |  | 0.015 |
| frameshift |  | 4 (7.4%) | 2 (6.5%) | 2 (8.7%) |  |
| in-frame deletion |  | 4 (7.4%) | 0 (0%) | 4 (17%) |  |
| large deletion |  | 16 (30%) | 12 (39%) | 4 (17%) |  |
| missense |  | 13 (24%) | 10 (32%) | 3 (13%) |  |
| nonsense |  | 11 (20%) | 3 (9.7%) | 8 (35%) |  |
| splice |  | 1 (1.9%) | 1 (3.2%) | 0 (0%) |  |
| NA |  | 5 (9.3%) | 3 (9.7%) | 2 (8.7%) |  |
|  |  |  |  |  |  |
| **Tissue Samples** | 130 |  |  |  | 0.8 |
| Tumor |  | 54 (41%) | 31 (42%) | 23 (41%) |  |
| NAT |  | 31 (24%) | 16 (22%) | 15 (27%) |  |
| PC |  | 45 (35%) | 27 (36%) | 18 (32%) |  |
| *^1^*Median (IQR); n (%) | | | | | |
| *^2^*Wilcoxon rank sum test; Pearson's Chi-squared test; Fisher's exact test | | | | | |

# Supplementary figures and legends


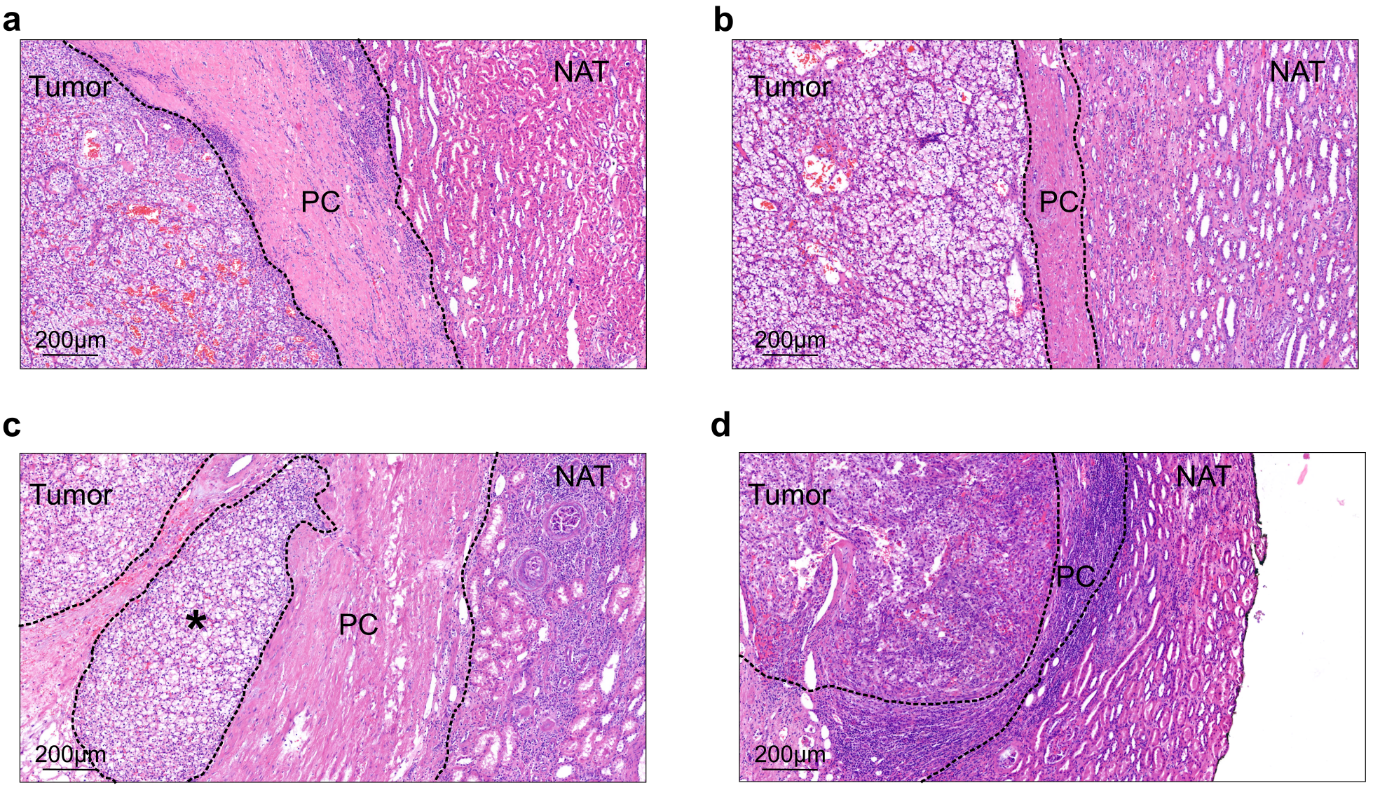


**Figure S1. Pseudocapsule composition:** (**a–b**) Representative H&E images of the ccRCC fibrous pseudocapsule (PC; dashed line), showing two phenotypes: (**a**) broad fibrous PC and (**b**) thin fibrous PC formed between the tumor and non-malignant adjacent tissue (NAT). (**c**) Exemplary micronodule formation (asterisk) within a thick fibrous PC. (**d**) Poorly defined PC with predominant lymphocytic infiltration.


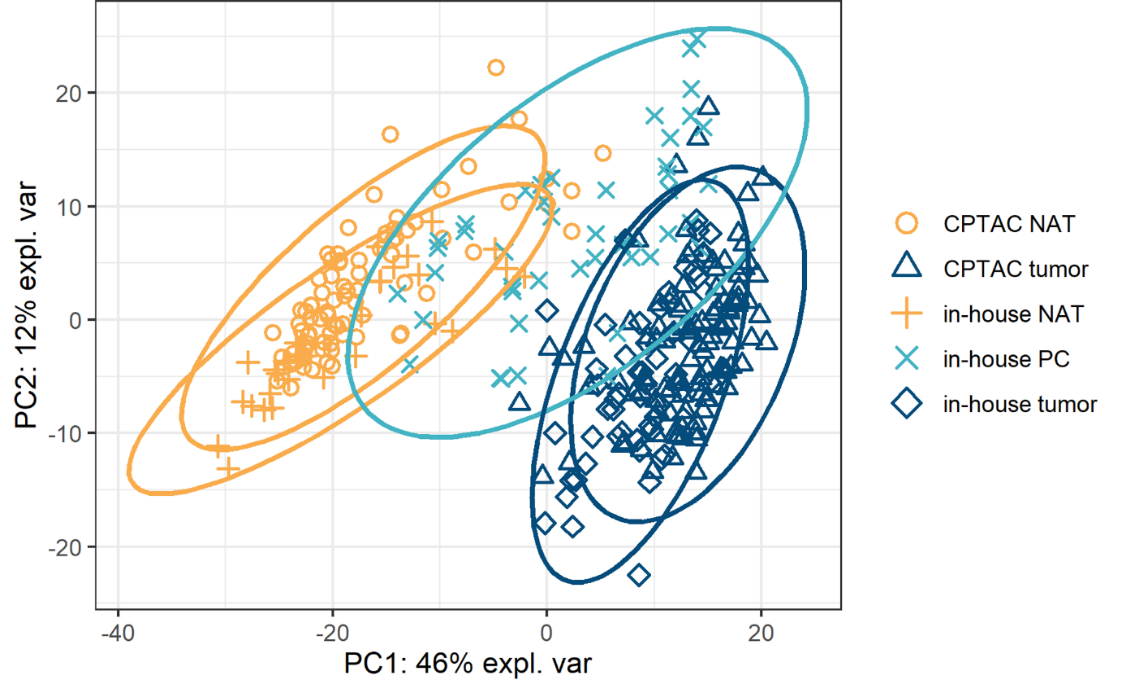


**Figure S2.** Principal component analysis (PCA) showing tumor, NAT, and PC samples of our in-house syndromic ccRCC and public CPTAC dataset. Ovals indicate 95% confidence intervals.


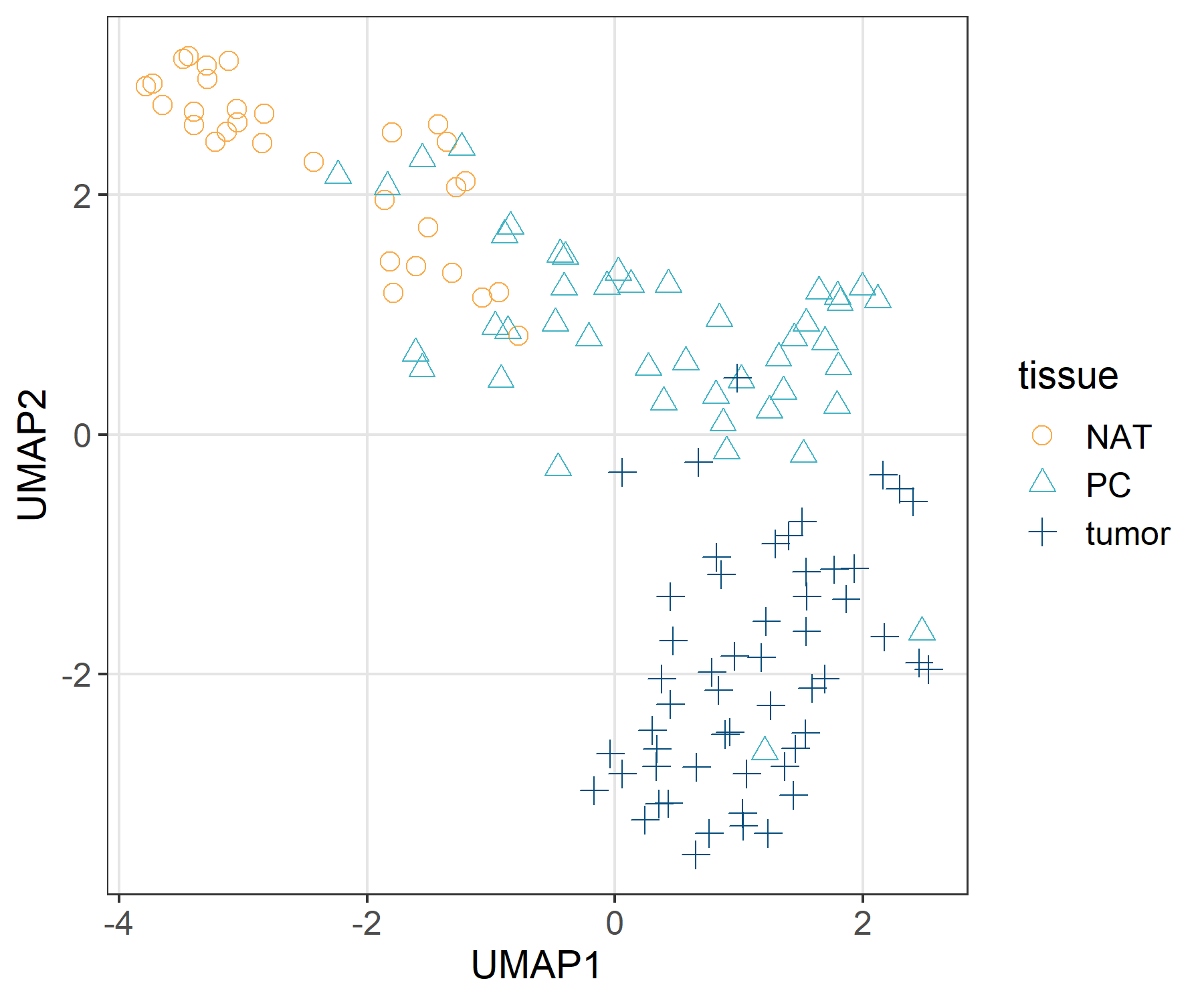


**Figure S3.** Uniform Manifold Approximation and Projection (UMAP) of tumor, PC, and NAT samples shows clear separation of tumor and NAT, with intermediate clustering of PC samples.


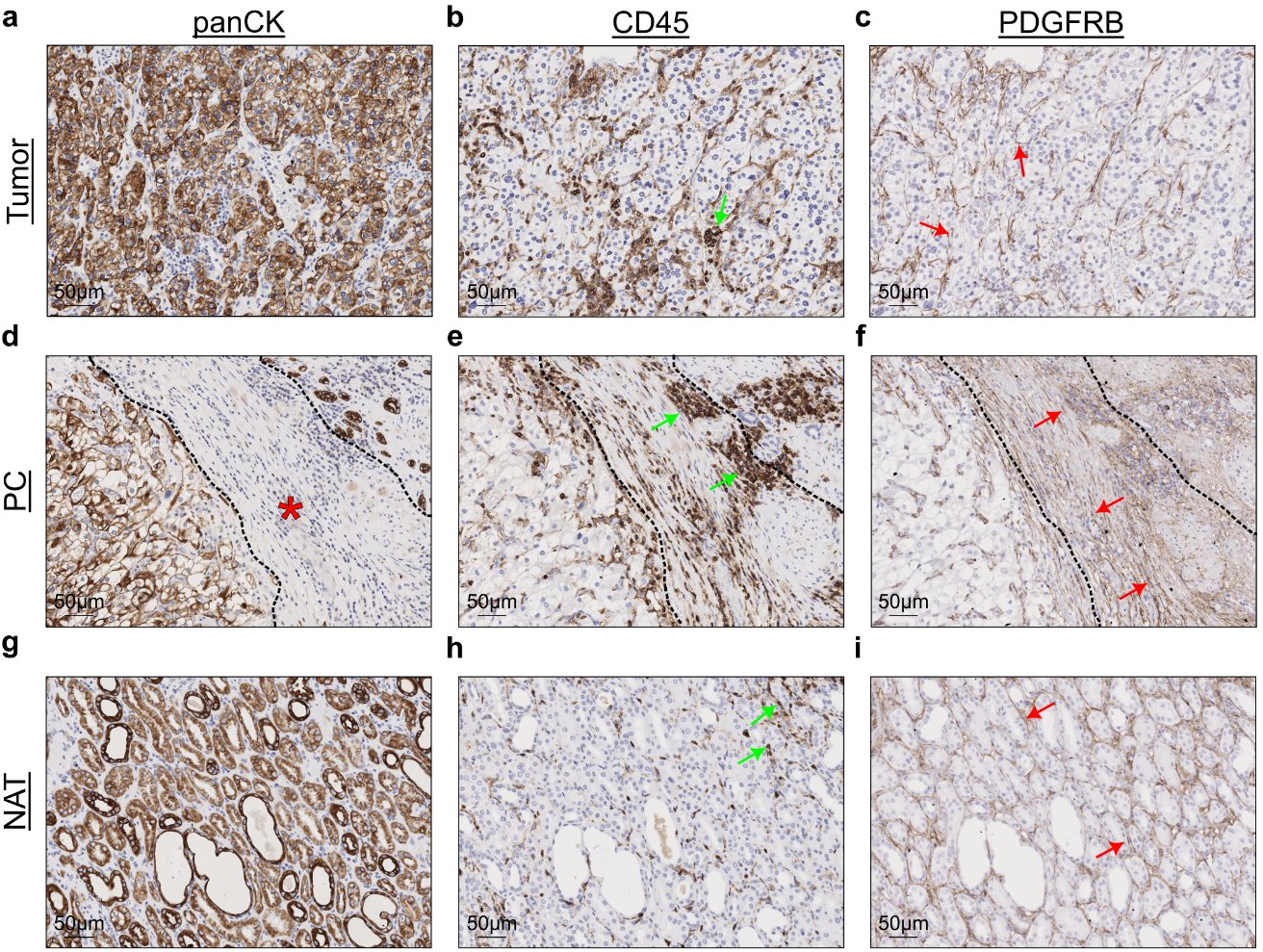


**Figure S4.** **Cellular Phenotypes in the ccRCC Compartments:** Representative IHC of tumor (a–c), fibrous capsule (d–f), and non-malignant adjacent tissue (g–i). Serial sections were stained for pan–cytokeratin (panCK; epithelial cells), CD45 (leukocytes; green arrows) and PDGFRβ (fibroblasts/interstitial cells; red arrows). In the tumor (a–c), panCK⁺ cells dominate, with scattered CD45⁺ and PDGFRβ⁺ cells in the intertumoral nests. Along the capsule (d–f; boundary marked by dashed line and asterisk), epithelial staining end at the border of the capsule and both CD45⁺ and PDGFRβ⁺ cells are dominant in this compartment. In NAT (g–i), panCK labels renal tubules, while CD45⁺ and PDGFRβ⁺ cells are infrequent.


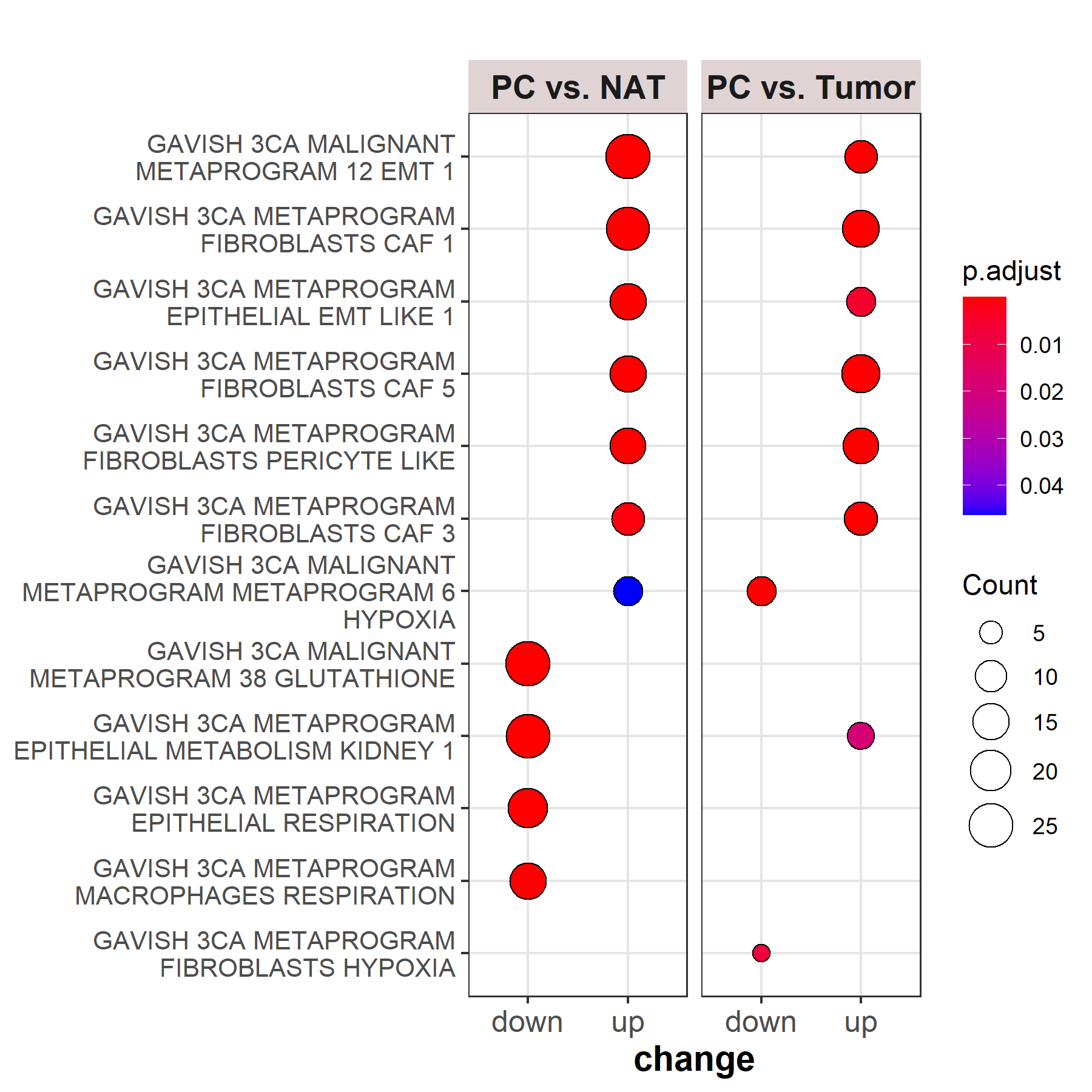


**Figure S5:** Curated Cancer Cell Atlas (3CA) gene set analysis demonstrates enrichment of processes linked to epithelial-mesenchymal transition (EMT) cancer-associated fibroblasts (CAF) is the PC. Compared to NAT, the PC shows depletion of metabolic processes and respiration, alongside increased hypoxia.


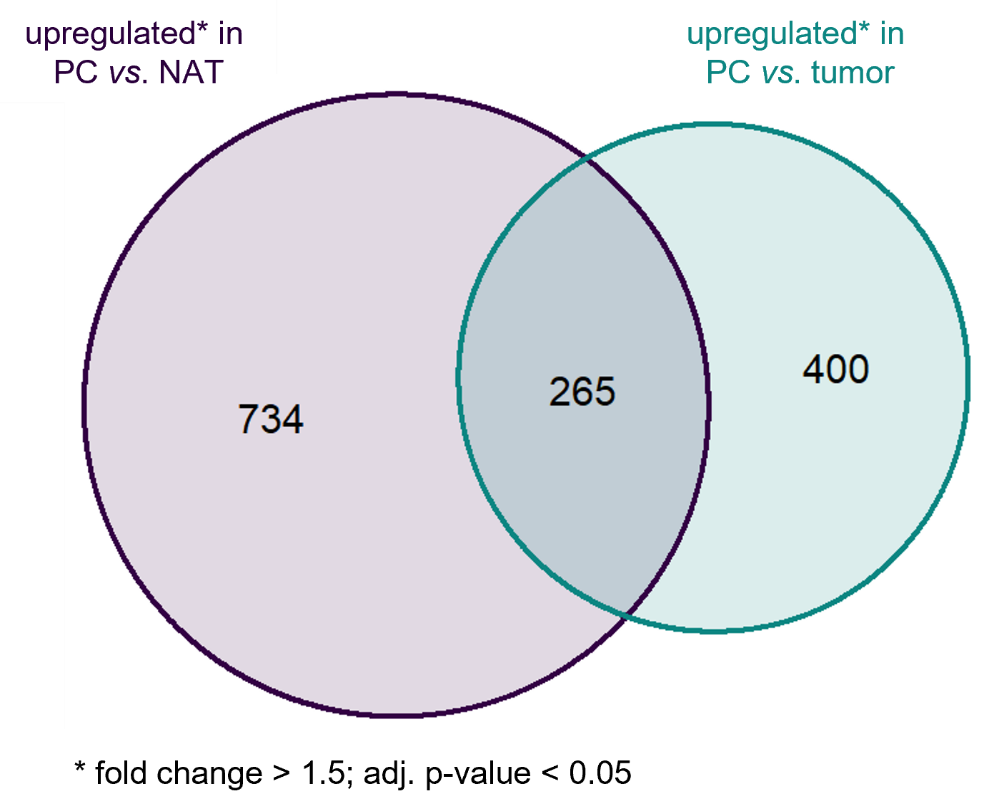


**Figure S6.** Venn diagram of upregulated proteins in PC vs. NAT and PC vs. tumor (fold change > 1.5, BH adj. p-value < 0.05).


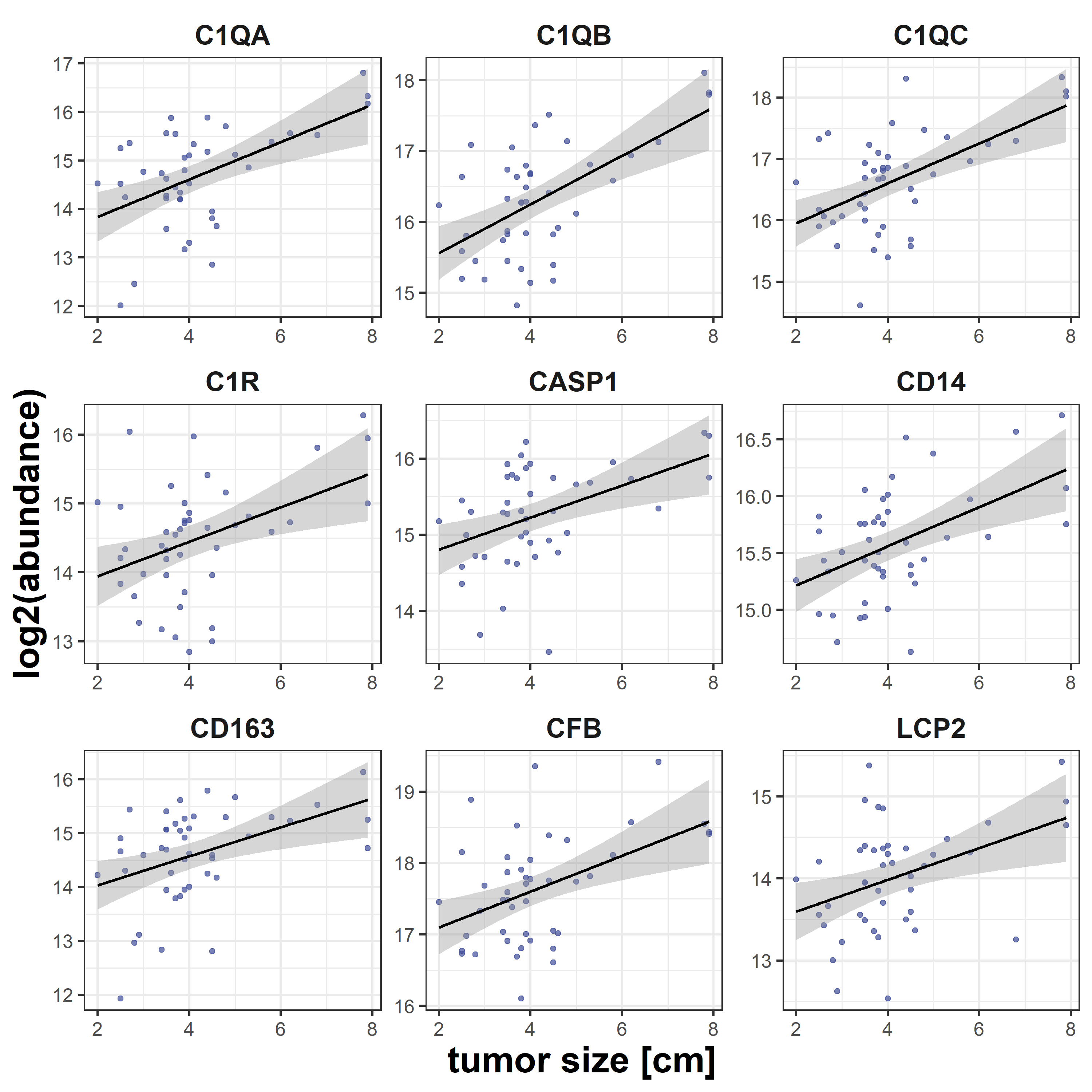


**Figure S7.** Scatter plots of selected PC proteins showing positive correlation with tumor size.


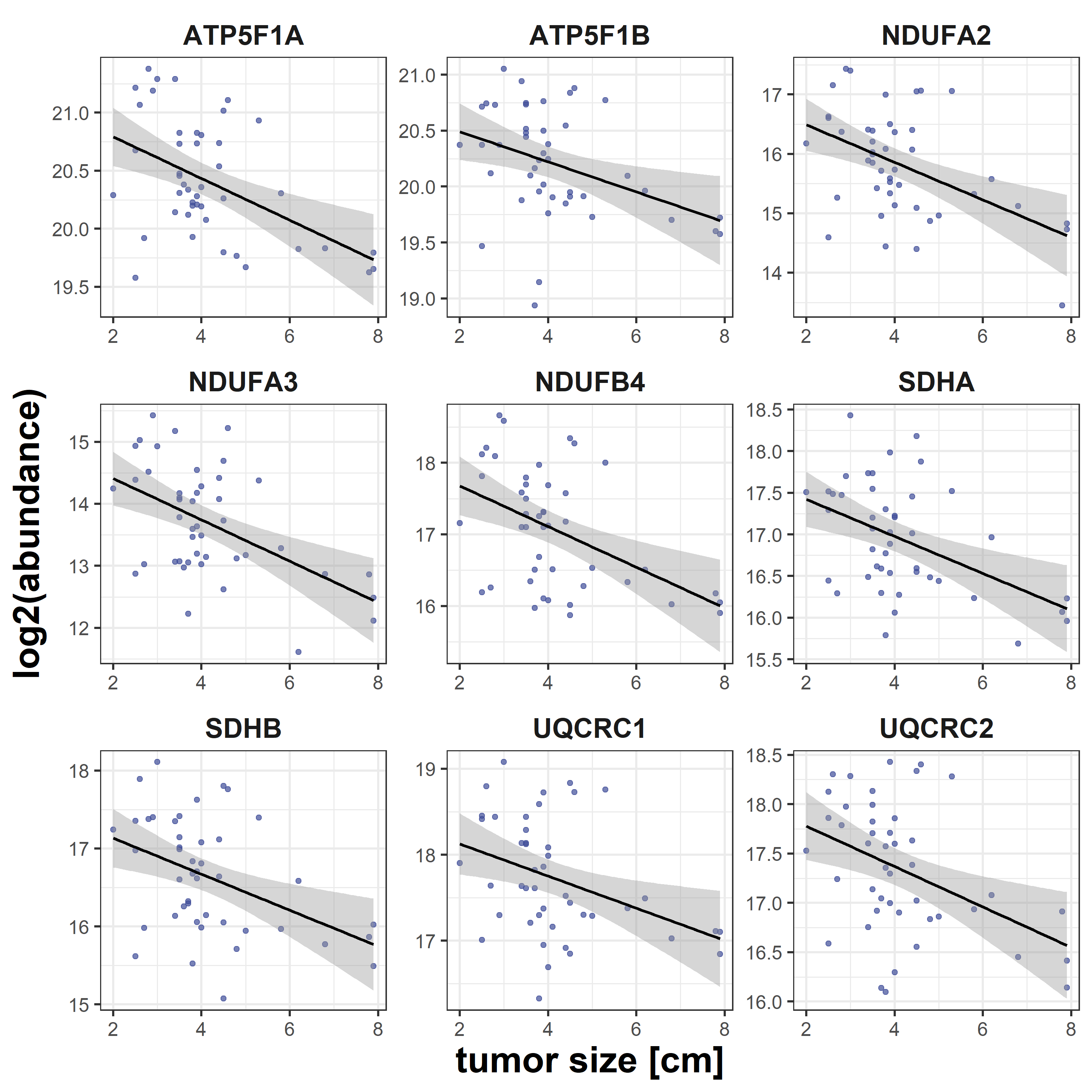


**Figure S8.** Scatter plots of selected PC proteins showing negative correlation with tumor size.


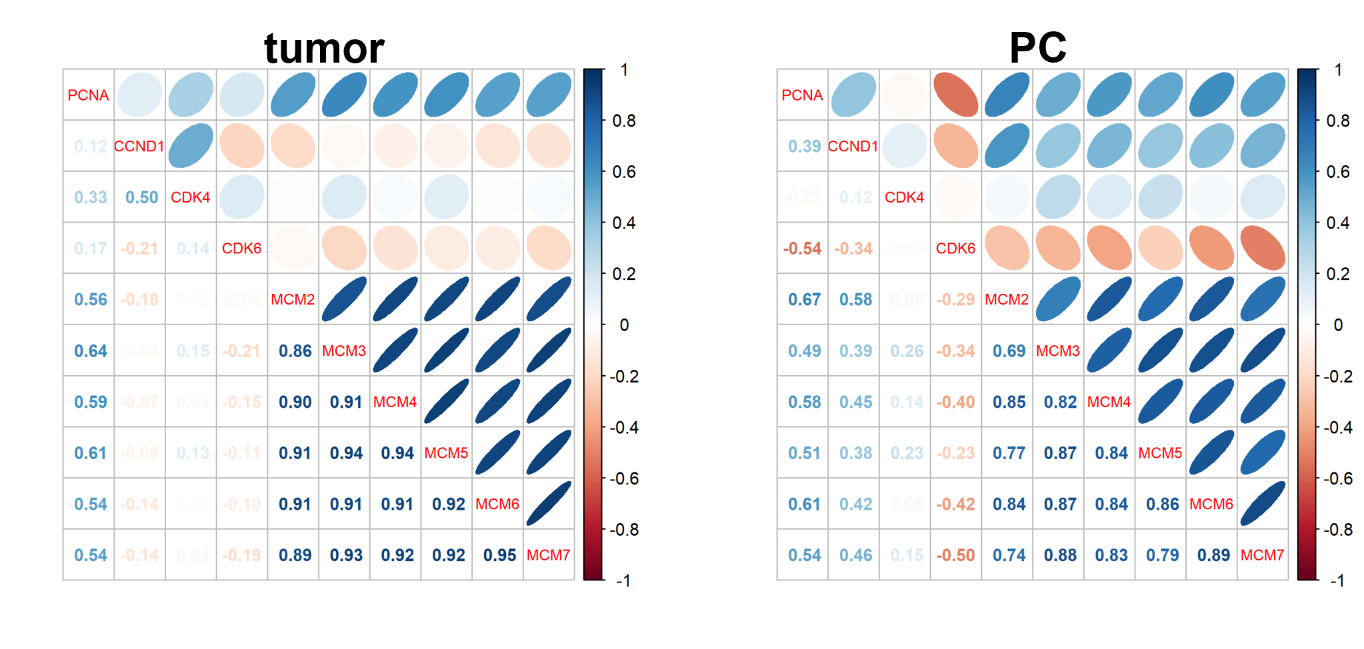


**Figure S9.** **Compartment-resolved correlations of proliferation markers in ccRCC:** Pearson correlation matrices depict pair-wise associations among the proliferation marker PCNA and canonical cell-cycle regulators (CCND1, CDK4, CDK6, MCM2–MCM7) tumor (left) and PC (right) samples. Ellipse orientation and color indicate the direction and strength of the correlation (blue = positive; red = negative). Numbers give the exact Pearson r values.


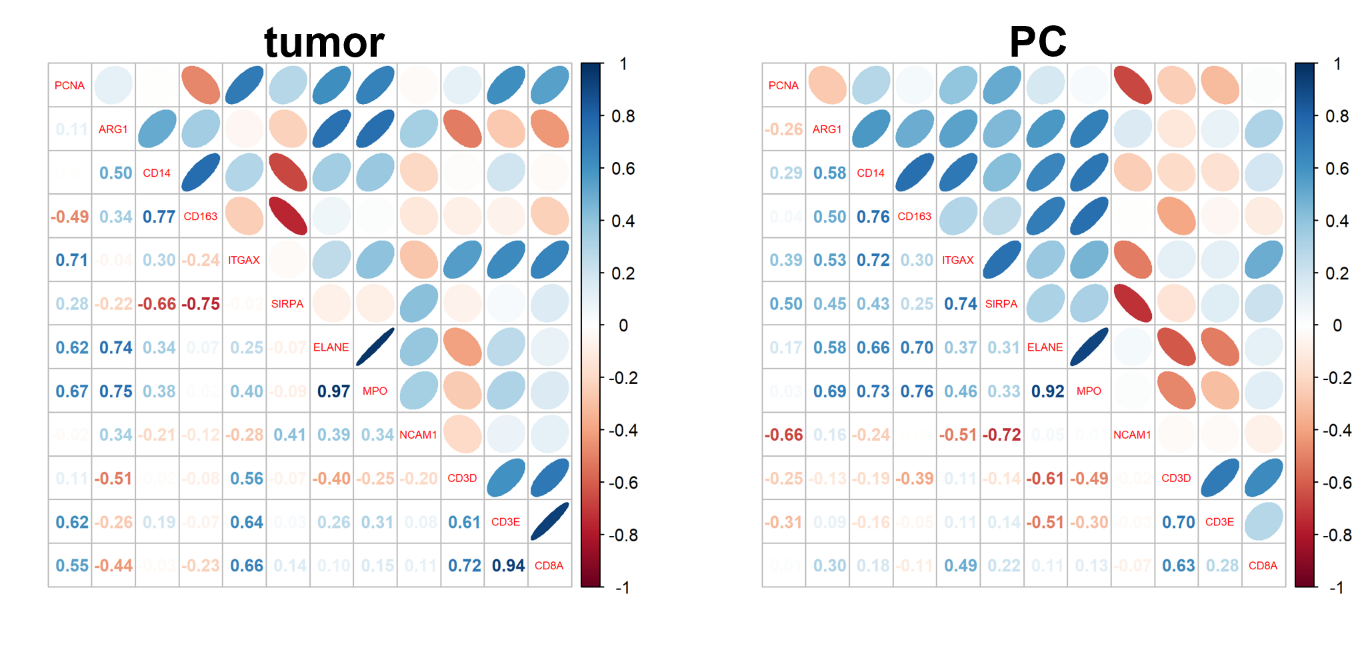


**Figure S10. Compartment-resolved correlations of immune markers in ccRCC:** Pearson correlation matrices compare the proliferation marker PCNA with a curated panel of immune-cell marker proteins (ARG1, CD14, CD163, ITGAX, SIRPA, ELANE, MPO, NCAM1, CD3D, CD3E, CD8A) in ccRCC tumor (left) and PC samples (right). Ellipse orientation and color indicate the direction and strength of the correlation (blue = positive; red = negative). Numbers give the exact Pearson r values.


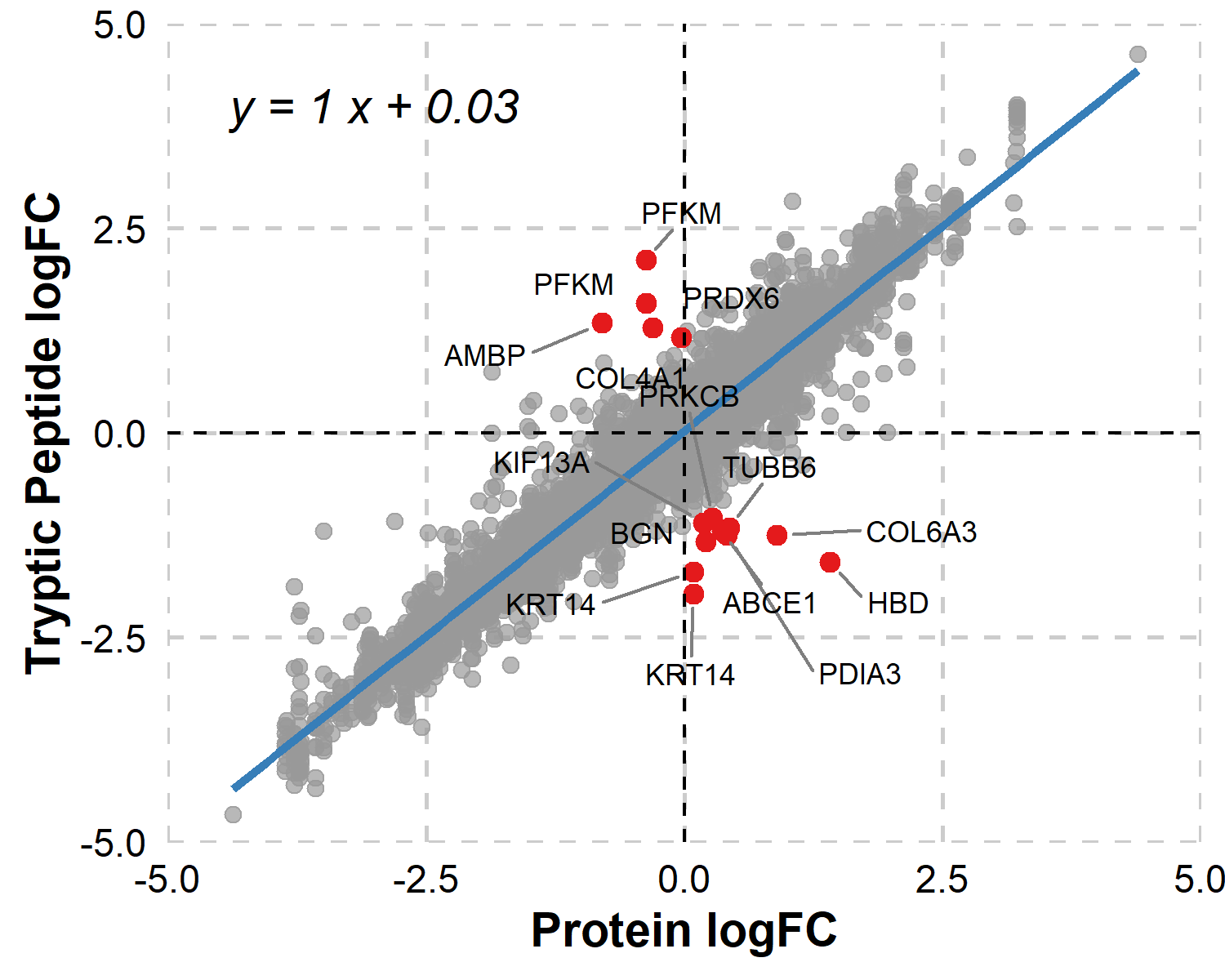


**Figure S11:** Scatter plot of tryptic peptide-level and protein-level log2 fold changes comparing tumor and NAT samples. Significantly changed peptides, where the corresponding proteins showed no or opposite regulation, are marked in red**.**


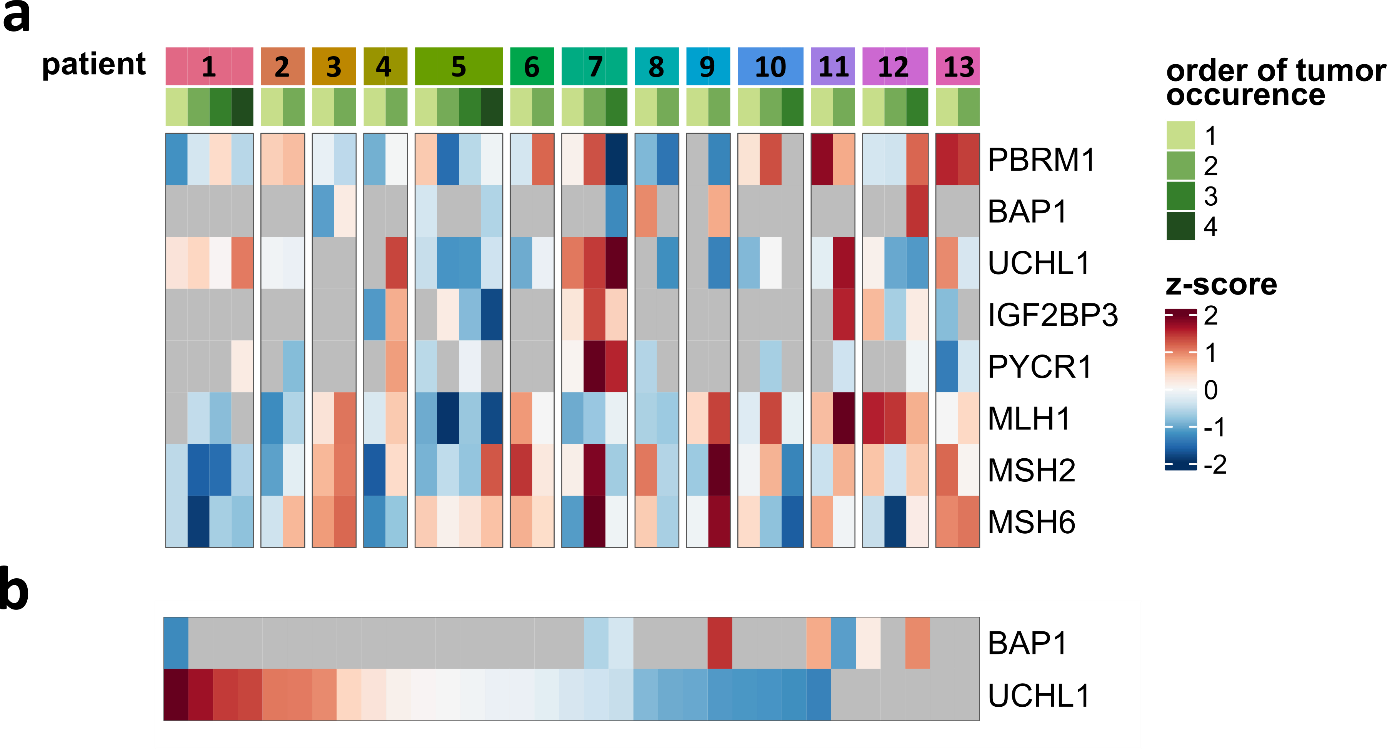


**Figure S12. a** Heatmap comparing the abundance of selected ccRCC markers, proteins associated with genome instability and DNA mismatch repair between primary and metachronous tumors. **b** Heatmap showing the abundance of BAP1 and UCHL1 in tumor samples. In tumors with lower or missing UCHL1 detection, BAP1 is detected more frequently.
